# Formation of hybrid higher-order cylindrical vector beams using binary multi-sector phase plates

**DOI:** 10.1038/s41598-018-32469-0

**Published:** 2018-09-25

**Authors:** Svetlana N. Khonina, Andrey V. Ustinov, Sergey A. Fomchenkov, Alexey P. Porfirev

**Affiliations:** 1 Samara National Research University, Technical Cybernetics Department, Samara, 443086 Russia; 2 Image Processing Systems Institute - Branch of the Federal Scientific Research Centre “Crystallography and Photonics” of Russian Academy of Sciences, Samara, 443001 Russia

## Abstract

Nowadays, the well-known cylindrical vector beams (CVBs) – the axially symmetric beam solution to the full-vector electromagnetic wave equation – are widely used for advanced laser material processing, optical manipulation and communication and have a great interest for data storage. Higher-order CVBs with polarisation order greater than one and superpositions of CVBs of various orders (hybrid CVBs) are especially of interest because of their great potential in contemporary optics. We performed a theoretical analysis of the transformation of first-order CVBs (radially and azimuthally polarised beams) into hybrid higher-order ones using phase elements with complex transmission functions in the form of the cosine or sine functions of the azimuthal angle. Binary multi-sector phase plates approximating such transmission functions were fabricated and experimentally investigated. The influence of the number of sectors and a height difference between neighbouring sectors, as well as the energy contribution of the different components in the generated hybrid higher-order CVBs were discussed in the context of polarisation transformation and vector optical field transformation in the focal region. The possibility of polarisation transformation, even in the case of weak focusing, is also demonstrated. The simple structure of the profile of such plates, their high diffraction efficiency and high damage threshold, as well as the easy-to-implement polarisation transformation principle provide advanced opportunities for high-efficient, quickly-switchable dynamic control of the generation of structured laser beams.

## Introduction

Along with full control of amplitude and phase distributions, control of the polarisation state of the generated laser radiation is crucial for applications in the field of optical manipulation^1,2^ and laser material processing^3–7^, for example, the use of heterogeneously polarised beams to shape matter with subwavelength features in a desired way^7^. The so-called cylindrical vector beams (CVBs), a class of axially symmetric laser beams with spatially variant polarisation^8^ also make it possible to realise a long-range tractor beam for airborne light-absorbing particles^9^ and to increase the capacity of free-space and fibre optical communication systems^10,11^. Due to this, paraxial and non-paraxial propagation of CVBs with polarisation singularities, including beams with radial, azimuthal and hybrid polarisation, is extensively studied^12–19^. Many studies on tight focusing of singular laser beams with complex types of polarisations have showed the possibility to use it for overcoming the diffraction limit^20,21^ as well as the three-dimensional control of a light field formed in the focal region^22–24^.

Inhomogeneously polarised beams can be generated using interferometric techniques^25^, anisotropic crystals^26–30^, subwavelength gratings^31–33^, *q*-plates^34^ and S-waveplates^35^. In two latter cases, both single plates and their cascades are widely used to generate higher-order cylindrical polarisation^36–39^. In fact, *q*-plates are subwavelength periodic structures behaving as a uniaxial crystal with the optical axes parallel and perpendicular to the subwavelength grooves. Such *q*-plates tuned by the temperature control^40^ or the external electric field^41,42^ can be used for efficient generation of higher-order CVBs at different wavelengths, however there are no tuned *q*-plates capable of performing the tunable generation of cylindrical polarisation of different orders. For this propose, optical setups with spatial light modulators (SLMs) supporting implementation of multi-level phase profile^43–46^ or a combination of such SLMs with a *q*-plate^47^ are usually used. Despite the advantages of using SLMs to perform dynamic control of the generated light fields, the limitation of this approach is well known, that is the relatively low damage threshold and efficiency of commercially available solutions, which somewhat limits the use of SLMs with high power lasers, for example, it requires additional SLM cooling systems^48^.

Previously, we demonstrated the conversion of an azimuthally polarised beam to a radially polarised beam, and vice versa, by introducing a higher-order vortex phase singularity into an investigated CVB^17^. In this paper, we demonstrate that interrelation of polarisation with the phase of the light field can be used for the transformation of an order of cylindrical polarisation and generation of hybrid CVBs and their superpositions. In contrast to the above-mentioned complex techniques, we propose to use easy-to-manufacture two-level pure-phase diffractive optical elements, the so-called binary multi-sector phase plates, fabricated on the fused silica substrate with a high damage threshold to realise the transformation of CVBs. The numerical simulation and experimental results obtained demonstrate the efficient formation of *n*th-order CVBs and their superpositions using *n*-sector phase plates even under conditions of weak focusing (NA < 0.7).

## Results

### Theoretical analysis

For the description of the focusing of CVBs in both the paraxial and non-paraxial cases, the Debye approximation is widely used^25^. In this case the electric field components of a monochromatic electromagnetic wave can be calculated as follows:

1 $$\begin{array}{rcl}{\bf{E}}(\rho ,\phi ,z) & = & (\begin{array}{c}{E}_{x}(\rho ,\phi ,z)\\ {E}_{y}(\rho ,\phi ,z)\\ {E}_{z}(\rho ,\phi ,z)\end{array})\\  & = & -\,\frac{if}{\lambda }\,{\int }_{0}^{\alpha }\,{\int }_{0}^{2\pi }\,(\begin{array}{cc}[1+{\cos }^{2}\,\varphi (\cos \,\theta -\mathrm{1)}] & \sin \,\varphi \,\cos \,\varphi (\cos \,\theta -\mathrm{1)}\\ \sin \,\varphi \,\cos \,\varphi (\cos \,\theta -\mathrm{1)} & [1+{\sin }^{2}\,\varphi (\cos \,\theta -\mathrm{1)}]\\ -\sin \,\theta \,\cos \,\varphi  & -\sin \,\theta \,\sin \,\varphi \end{array})(\begin{array}{c}{c}_{x}(\varphi )\\ {c}_{y}(\varphi )\end{array})\\  &  & \times \,B(\theta ,\varphi )T(\theta )\,\exp [ik(\rho \,\sin \,\theta \,\cos (\varphi -\phi )+z\,\cos \,\theta )]\,\sin \,\theta d\theta d\varphi ,\end{array}$$

where (*ρ*, *φ*, *z*) are the cylindrical coordinates in the focal region, (*θ*, *ϕ*) are the spherical angular coordinates of the focusing system’s output pupil, *α* is the maximum value of the azimuthal angle related to the system’s numerical aperture (NA), so changing the value *α* makes it possible to vary the sharpness of focusing, *B*(*θ*, *ϕ*) is the transmission function, *T*(*θ*) is the pupil’s apodization function (equal to $\sqrt{\cos \,\theta }$ for aplanatic systems), *k* = 2*π*/*λ* is the wavenumber, *λ* is the wavelength, *f* is the focal length, *c*_*x*_(*ϕ*) and *c*_*y*_(*ϕ*) are the polarisation coefficients of the incident radiation. It is evident that for small values of NA, the *z*-component of the electric field becomes insignificant.

Different types of CVBs with the polarisation order *p* and the inner polarisation rotation of the beam *ϕ*_0_ can be described by the following generalised expression^36^:

2 $${\bf{C}}(\varphi )=(\begin{array}{c}\cos (p\varphi +{\varphi }_{0})\\ \sin (p\varphi +{\varphi }_{0})\end{array})\mathrm{.}$$

For light fields described by Eq. (2) and for radially-symmetric light fields (*B*(*θ*, *ϕ*) = *R*(*θ*)), Eq. (1) can be written as follows:

3 $${{\bf{E}}}_{p}(\rho ,\phi ,z)=-\,ikf\,{\int }_{0}^{\alpha }\,R(\theta )T(\theta ){{\bf{Q}}}_{p}(\rho ,\phi ,\theta )\,\sin \,\theta \,\exp (ikz\,\cos \,\theta )d\theta ,$$

where

4 $$\begin{array}{rcl}{{\bf{Q}}}_{p}(\rho ,\phi ,\theta ) & = & {i}^{p}(\begin{array}{c}{J}_{p}(t)\,\cos (p\phi +{\varphi }_{0})-\frac{1}{2}\{{J}_{p}(t)\,\cos (p\phi +{\varphi }_{0})-{J}_{p-2}(t)\,\cos \,[(p-\mathrm{2)}\phi +{\varphi }_{0}]\mathrm{\}(1}-\,\cos \,\theta )\\ {J}_{p}(t)\,\sin (p\phi +{\varphi }_{0})-\frac{1}{2}\{{J}_{p}(t)\,\sin (p\phi +{\varphi }_{0})+{J}_{p-2}(t)\,\sin [(p-\mathrm{2)}\phi +{\varphi }_{0}]\mathrm{\}(1}-\,\cos \,\theta )\\ i{J}_{p-1}(t)\,\cos [(p-\mathrm{1)}\phi +{\varphi }_{0}]\,\sin \,\theta \end{array}),\end{array}$$

with *t* = *kρ* sin *θ*.

**Figure 1:**
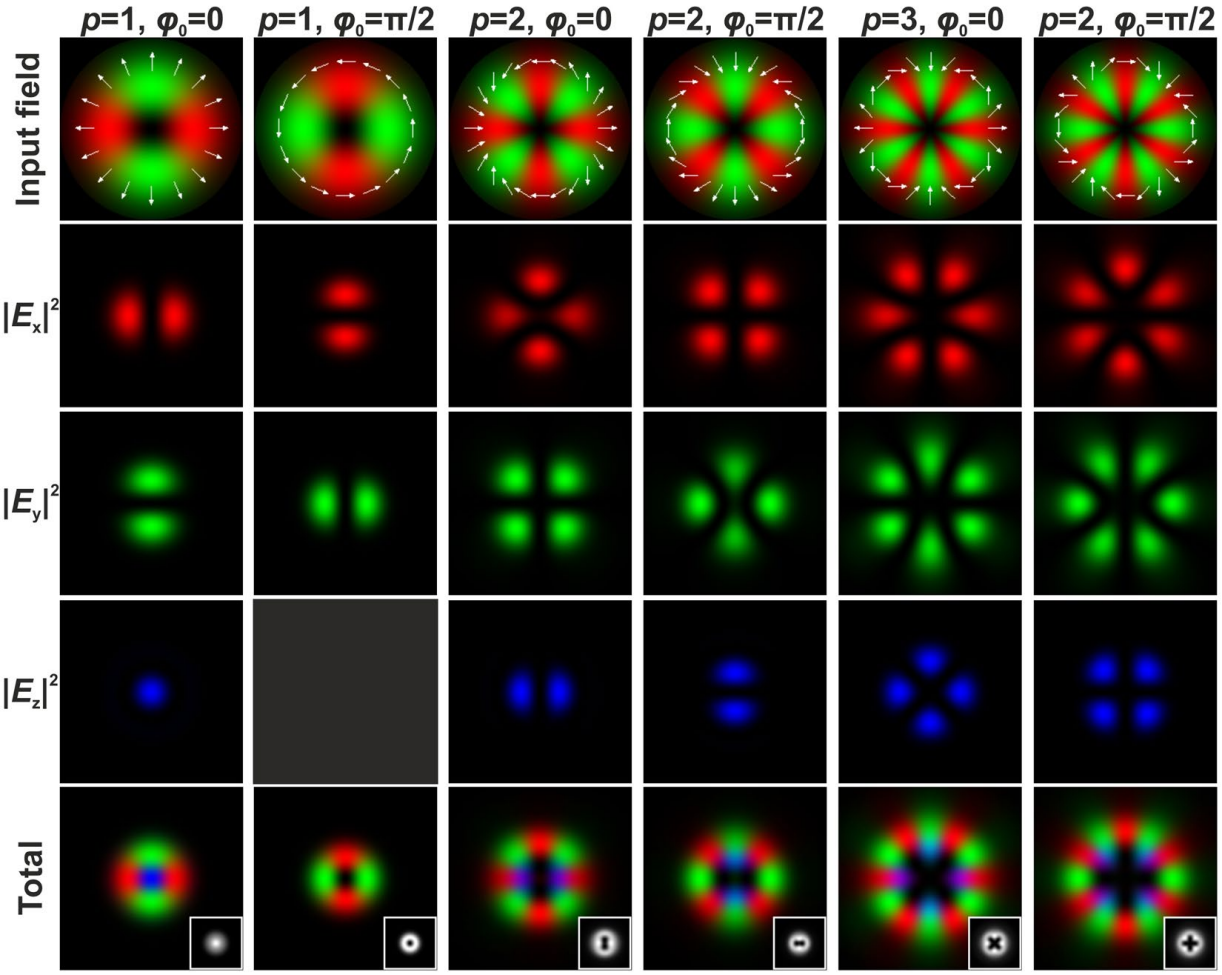
The distribution of the various components of the electric field in the focal plane of a focusing system (NA = 0.99) for an incident CVB with different positive polarisation order *p* and different inner polarisation rotation of the beam *ϕ*_0_. The insets in the total field distribution images show the generated grayscale intensity distributions. The white arrows show the schematic polarisation distributions.

**Figure 2:**
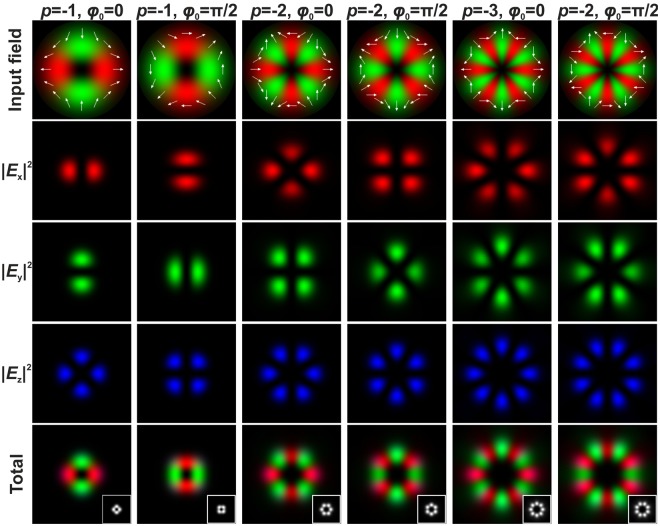
The distribution of the various components of the electric field in the focal plane of a focusing system (NA = 0.99) for an incident CVB with different negative polarisation order *p* and different inner polarisation rotation of the beam *ϕ*_0_. The insets in the total field distribution images show the generated grayscale intensity distributions. The white arrows show the schematic polarisation distributions.

As follows from Eq. (4), the *z*-component of a tightly focused electromagnetic field is not zero at the optical axis only in the case when *p* = 1, and *ϕ*_0_ = 0 or *π*, that is, in the case of radial polarisation. The *z*-component completely disappears in the case when *p* = 1, *ϕ*_0_ = *π*/2 or 3*π*/2, that is, in the case of azimuthal polarisation. For other cases of CVBs, the *z*-component has cosine or sine dependence on the angle *φ*: for example, for a CVB with *p* = −1 and *ϕ*_0_ = 0 *E*_*z*_(*ρ*, *φ*, *z*) ∝ cos(2*φ*) and for a CVB with *p* = −1 and *ϕ*_0_ = *π*/2 *E*_*z*_(*ρ*, *φ*, *z*) ∝ sin(2*φ*) (see Figs 1 and 2). Such negative-order CVBs can be obtained by passing the positive-order CVB through a half waveplate^49–52^. In the case of negative-order CVBs, light field patterns generated after passing through a rotating linear polariser rotate in the direction opposite that of rotation in the case of positive-order CVBs^49^. In contrast to the positive-order CVBs, the energy contribution of the formed *z*-component of the tightly focused negative-order CVBs is always less than the energy contribution of the *x*- and *y*-components (see Fig. 2).

### Generation of hybrid CVBs using multi-sector phase plates

The interrelation of polarisation with the phase of light field allows the use of diffractive optical elements for the transformation of first-order or lower-order CVBs into higher-order CVBs (*p* > 1). The easiest way to increase the polarisation order is to increase the multiplicity of the angle in Eq. (2). A partial solution of this problem is possible by multiplying the initial cylindrically polarised electromagnetic field by the cosine or sine function of a multiple angle. For example, a combination of the radially polarised beam with the cosine function of the angle *φ* has the following form:

5 $${{\bf{e}}}_{Rad}\,\cos \,\phi =(\begin{array}{c}\cos \,\phi \\ \sin \,\phi \end{array})\,\cos \,\phi =\frac{1}{2}(\begin{array}{c}1+\,\cos \,2\phi \\ \sin \,2\phi \end{array})=\frac{1}{2}(\begin{array}{c}1\\ 0\end{array})+\frac{1}{2}(\begin{array}{c}\cos \,2\phi \\ \sin \,2\phi \end{array})=\frac{1}{2}{{\bf{e}}}_{Lin\_x}+\frac{1}{2}{{\bf{e}}}_{p=2,{\varphi }_{0}=0}.$$

It is clear that the result of Eq. (5) is in fact a superposition of two laser beams - a beam linearly polarised in the *x*-direction and a second-order CVB. For a combination of the first-order radial polarisation with the sine function of the angle *φ*, the following result is obtained:

6 $${{\bf{e}}}_{Rad}\,\sin \,\phi =(\begin{array}{c}\cos \,\phi \\ \sin \,\phi \end{array})\,\sin \,\phi =\frac{1}{2}(\begin{array}{c}\sin \,2\phi \\ 1-\,\cos \,2\phi \end{array})=\frac{1}{2}(\begin{array}{c}0\\ 1\end{array})-\frac{1}{2}(\begin{array}{c}-\sin \,2\phi \\ \cos \,2\phi \end{array})=\frac{1}{2}{{\bf{e}}}_{Lin\_y}-\frac{1}{2}{{\bf{e}}}_{p=2,{\varphi }_{0}=\pi /2},$$

that is, a superposition of two laser beams with polarisations orthogonal to those obtained in Eq. (5), namely, a beam linearly polarised in the *y*-direction and a second-order CVB with the inner polarisation rotation of *π*/2.

In general, an increase in the multiplicity of the angle for the used cosine and sine functions leads to the following transformation:

7 $$\begin{array}{rcl}{{\bf{e}}}_{Rad}\,\cos \,m\phi  & = & \frac{1}{2}{{\bf{e}}}_{p=-(m-1),{\varphi }_{0}=0}+\frac{1}{2}{{\bf{e}}}_{p=(m+1),{\varphi }_{0}=0},\\ {{\bf{e}}}_{Rad}\,\sin \,m\phi  & = & \frac{1}{2}{{\bf{e}}}_{p=-(m-1),{\varphi }_{0}=\pi /2}-\frac{1}{2}{{\bf{e}}}_{p=(m+1),{\varphi }_{0}=\pi /2}\mathrm{.}\end{array}$$

Thus, superpositions of higher-order CVBs are formed.

For a case of using the azimuthally polarised laser beam as an initial beam, the following expressions are obtained:

8 $$\begin{array}{rcl}{{\bf{e}}}_{Az}\,\cos \,\phi  & = & (\begin{array}{c}-\sin \,\phi \\ \cos \,\phi \end{array})\,\cos \,\phi =\frac{1}{2}{{\bf{e}}}_{Lin\_y}+\frac{1}{2}{{\bf{e}}}_{p=2,{\varphi }_{0}=\pi /2},\\ {{\bf{e}}}_{Az}\,\sin \,\phi  & = & (\begin{array}{c}-\sin \,\phi \\ \cos \,\phi \end{array})\,\sin \,\phi =-\,\frac{1}{2}{{\bf{e}}}_{Lin\_x}+\frac{1}{2}{{\bf{e}}}_{p=2,{\varphi }_{0}=0},\\ {{\bf{e}}}_{Az}\,\cos (m\phi ) & = & \frac{1}{2}{{\bf{e}}}_{p=-(m-1),{\varphi }_{0}=\pi /2}+\frac{1}{2}{{\bf{e}}}_{p=(m+1),{\varphi }_{0}=\pi /2},\\ {{\bf{e}}}_{Az}\,\sin (m\phi ) & = & -\,\frac{1}{2}{{\bf{e}}}_{p=-(m-1),{\varphi }_{0}=0}+\frac{1}{2}{{\bf{e}}}_{p=(m+1),{\varphi }_{0}=0}\mathrm{.}\end{array}$$

The well known binary multi-sector phase plates^53,54^ can be used for generation of functions approximating the above considered trigonometric functions^55–57^. The expansion in a Fourier series of the transmission function of a phase plate *f*(*φ*) = exp[*iψ*(*φ*)], where *ψ*(*φ*) is a phase of the phase plate, has the following form:

9 $$f(\phi )=\frac{{a}_{0}}{2}+\sum _{n=1}^{\infty }\,[{a}_{n}\,\cos (n\phi )+{b}_{n}\,\sin (n\phi )],$$

where

10 $$\begin{array}{l}{a}_{0}=\frac{1}{\pi }\,{\int }_{0}^{2\pi }\,f(\phi )d\phi ,\\ {a}_{n}=\frac{1}{\pi }\,{\int }_{0}^{2\pi }\,f(\phi )\,\cos (n\phi )d\phi ,\\ {b}_{n}=\frac{1}{\pi }\,{\int }_{0}^{2\pi }\,f(\phi )\,\sin (n\phi )d\phi .\end{array}$$

Note the contribution to the intensity near the optical axis comes from terms with the low indices *n*^56,57^; other terms will change just the off-axis distribution because of higher frequency.

Several configurations of the binary phase plates will now be considered.

1. ***Two-sector binary phase plate with phase values of φ***_**1**_ ***and φ***_**2**_ ***for different sectors***
  In this case, Eq. (10) is transformed to the following:
  11 $$\begin{array}{rcl}{a}_{0} & = & \exp (i{\phi }_{1})+\exp (i{\phi }_{2}),\\ {a}_{n} & = & 0,\\ {b}_{n} & = & (\begin{array}{ll}2[\exp (i{\phi }_{1})-\exp (i{\phi }_{2})]/(\pi n), & {\rm{for}}\,{\rm{odd}}\,n,\\ 0, & {\rm{for}}\,{\rm{even}}\,n.\end{array}\end{array}$$
  Then, the transmission function of the phase plate has the following form:
  12 $$f(\phi )=\frac{\exp (i{\phi }_{1})+\exp (i{\phi }_{2})}{2}+\frac{2}{\pi }[\exp (i{\phi }_{1})-\exp (i{\phi }_{2})]\,\sum _{n=0}^{\infty }\,\frac{\sin \,[(2n+1)\phi ]}{2n+1}.$$
  From Eq. (12), it is evident that such phase plate simultaneously generates a set of trigonometric functions with decreasing weight factors. In this case, if *φ*_1_ − *φ*_2_ = *π*, then the free term *a*_0_ is absent. In particular, when *φ*_1_ = 0 and *φ*_2_ = *π*, Eq. (12) is transformed to the following:
  13 $$f(\phi )=\frac{4}{\pi }\,\sum _{n=0}^{\infty }\,\frac{\sin \,[(2n+1)\phi ]}{2n+1}=\frac{4}{\pi }\,[\sin \,\phi +\frac{\sin (3\phi )}{3}+\frac{\sin (5\phi )}{5}+\ldots ],$$
  that is, the phase plate corresponds to a sum of the sine functions of the odd orders. Taking into account the decreasing weight factors, as well as the concentration of the energy in the focal region near the optical axis, we can assume that such a phase plate substantially corresponds to the sin *φ* function and can be used for transformations described by Eqs. (5)–(8). It is evident, when the phase plate rotates by 90 degrees, we get an analogous sum of cosine functions with cos *φ* as the main term.
2. ***N***-***sector binary phase plate with phase values of φ***_**1**_ ***and φ***_**2**_ ***for different sectors***

The general formula for an *N*-sector binary phase plate has the following form:

14 $$f(\phi )=\frac{\exp (i{\phi }_{1})+\exp (i{\phi }_{2})}{2}+\frac{2N}{\pi }\,[\exp (i{\phi }_{1})-\exp (i{\phi }_{2})]\,\sum _{n=0}^{\infty }\,\frac{\sin \,[(2n+1)N\phi ]}{(2n+1)N}.$$

As follows from Eq. (14), the first term of the series corresponds to the sin(*Nφ*) function, while the other harmonics have orders changing by 2*N* and proportionally decreasing weight factors. The presence of the free term (for *φ*_1_ − *φ*_2_ ≠ *π*) leads to the presence of an additional term corresponding to the initial polarisation state in the polarisation superpositions described by Eqs. (5)–(8). In order to estimate the energy contribution of the free term and the first (main) term in the series, we assume that *φ*_1_ = 0, then the energy contribution of the free term equals $|{a}_{0}{|}^{2}=4\,{\cos }^{2}({\phi }_{2}/2)$, and the energy contribution of the main term is $|{b}_{1}{|}^{2}=\frac{16}{{\pi }^{2}}\,{\sin }^{2}({\phi }_{2}/2)$. Thus, it is possible to change the ratio between the initial polarisation and the formed one by varying the value *φ*_2_. In particular, the equal energy contributions of these two components is when ${\phi }_{2}=2\,\arctan (\frac{\pi }{2\sqrt{2}})\approx 96^\circ$. It is evident that the average value of ${\sin }^{2}(N\phi )$ equal to 0.5 does not change when *N* is replaced with a multiple of it, so the total fraction of the total sum in Eq. (14) is $\frac{16}{{\pi }^{2}}{\sin }^{2}({\phi }_{2}/2)\frac{1}{2}(1+\frac{1}{{3}^{2}}+\frac{1}{{5}^{2}}+\cdots )={\sin }^{2}({\phi }_{2}/2)$, taking into account that the sum of the series in brackets is *π*^2^/8. Thus, the energy contributions of the free term and the total sum of the series are the same when *φ*_2_ = 90° = *π*/2.

**Figure 3:**
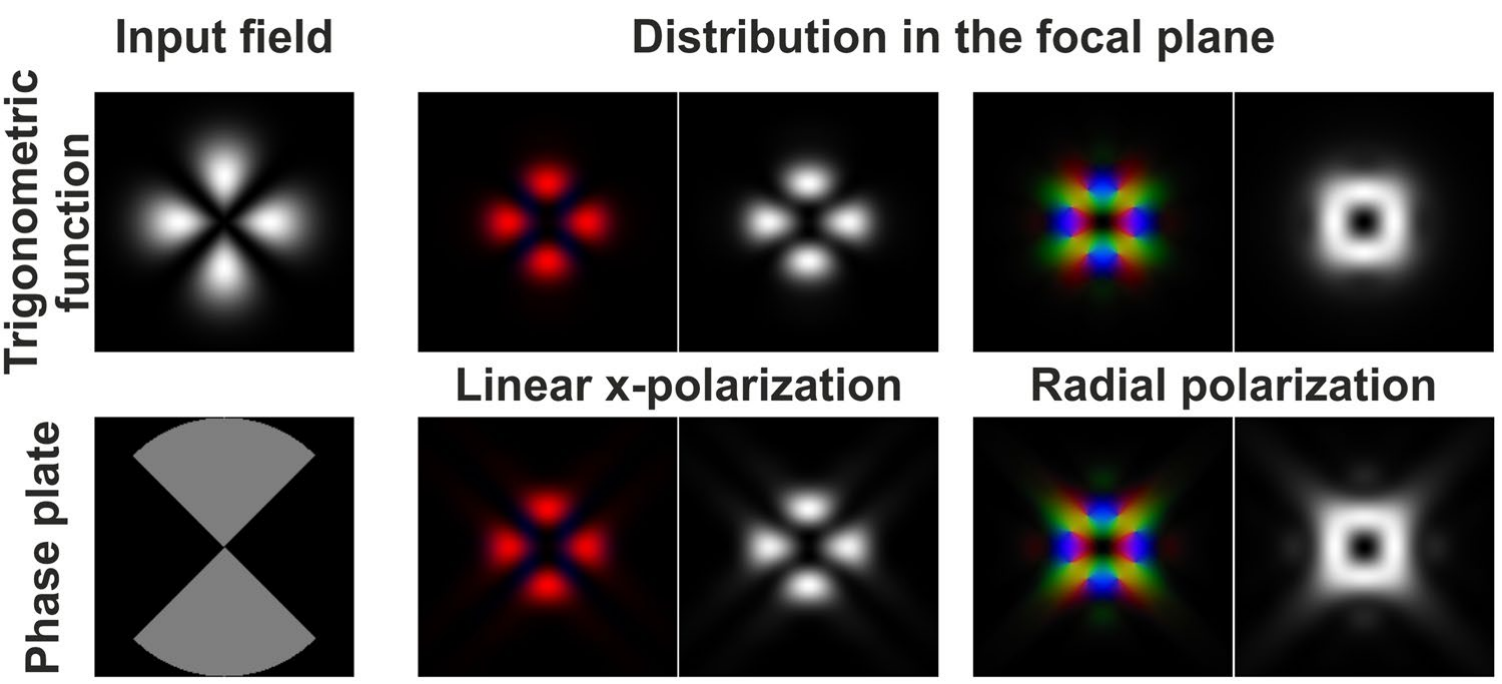
Comparison of the focusing of a Gaussian beam with linear polarisation and radial polarisation when the beam is multiplied by the cos(2*φ*) function (upper row) and when it passes through the binary plate in the form of *f*(*φ*) = exp{*iπ*(1 + sgn[cos(2*φ*)])/2} (bottom row).

Figure 3 visualises the results of replacing the trigonometric functions cos(*mφ*) or sin(*mφ*) by a binary multi-sector phase plate. The distributions formed in the focal region are very similar in structure. Some differences are observed only in the peripheral area^56^.

### Numerical modelling

**Figure 4:**
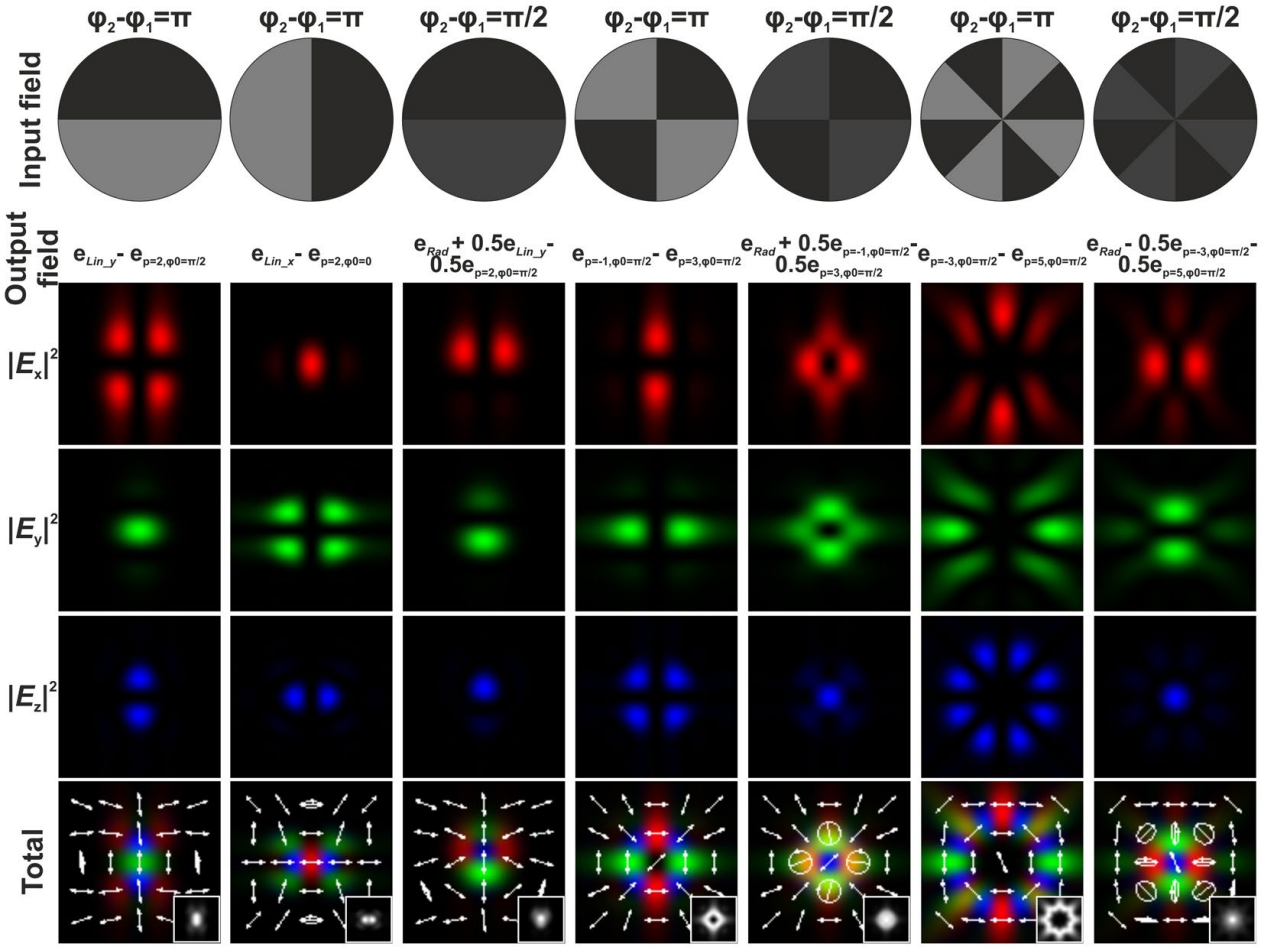
Transformation of the initial radially polarised laser beam passed through different binary multi-sector phase plates. In fact, the generated output fields are superpositions of different CVBs. The distribution of the various components of the electric field in the focal plane of a focusing system (NA = 0.99) are shown. The insets in the total field distribution images show the generated grayscale intensity distributions. The white arrows show the schematic polarisation distributions.

Figure 4 shows the modelling results for tight focusing (NA = 0.99) of a laser beam with radial polarisation passing through binary multi-sector phase plates with *φ*_1_ = 0, and *φ*_2_ = *π* or *π*/2. It is clear that the modelling results are in good agreement with the presented theoretical analysis. In the case of a two-sector phase plate with *φ*_2_ = *π* (the first column of Fig. 4), whose action analogous to the action of the sin *φ* function, an initial radially polarised CVB is transformed into a sum of a beam linearly polarised in the *y* direction ${{\bf{e}}}_{Lin\_y}$ and a second-order CVB ${{\bf{e}}}_{p=2,{\varphi }_{0}=\pi /2}$. When comparing the individual field components, it is evident that the *x*- and *z*-components correspond to the second-order CVB ${{\bf{e}}}_{p=2,{\varphi }_{0}=\pi /2}$ (see Fig. 1), and the *y*-component substantially consists of linear *y*-polarisation, because it is much larger than the corresponding component ${{\bf{e}}}_{p=2,{\varphi }_{0}=\pi /2}$. When this phase plate is rotated 90 degrees (the second column of Fig. 4), its action is similar to that of the cos(*φ*) function – that is, a superposition of laser beams with different polarisations (a linearly x-polarised beam ${{\bf{e}}}_{Lin\_x}$ and the second-order CVB ${{\bf{e}}}_{p=2,{\varphi }_{0}=0}$) is generated. As follows from Eq. (14) and the modelling results, the presence of the two-sector phase plate with *φ*_2_ = *π*/2 (the third column of Fig. 4) leads to a situation when half of the initial light energy does not change its initial polarisation state (radially polarised beam ${{\bf{e}}}_{Rad}$) and half of the initial light energy is transformed to a superposition of a linearly *y*-polarised beam and the second-order CVB ${{\bf{e}}}_{p=2,{\varphi }_{0}=0}$.

A four-sector phase plate with *φ*_2_ = *π* (the fourth column of Fig. 4), whose action is analogous to the sin(2*φ*) function, allows to generate the superposition of CVBs: $0.5{{\bf{e}}}_{p=-1,{\varphi }_{0}=\pi /2}-0.5{{\bf{e}}}_{p\mathrm{=3,}{\varphi }_{0}=\pi \mathrm{/2}}$. In this case, the longitudinal component of the generated superposition coincides with the CVB with *p* = −1 and *ϕ*_0_ = *π*/2. The presence of a four-sector phase plate with *φ*_2_ = *π*/2 (the fifth column of Fig. 4) leads to a superposition of the three CVBs $0.5{{\bf{e}}}_{Rad}+0.25{{\bf{e}}}_{p=-1,{\varphi }_{0}=\pi /2}+0.25{{\bf{e}}}_{p\mathrm{=3,}{\varphi }_{0}=\pi \mathrm{/2}}$. In this case, the central part of the generated light pattern has circular polarisation and the peripheral part of the pattern has the polarisation analogous to the polarisation formed by the 4-sector phase plate with *φ*_2_ = *π*. Finally, the presence of a 8-sector phase plate with *φ*_2_ = *π* or *π*/2 (the sixth and seventh columns of Fig. 4) leads to the superpositions of CVBs in the form of $0.25{{\bf{e}}}_{p=-3,{\varphi }_{0}=\pi /2}-0.25{{\bf{e}}}_{p=5,{\varphi }_{0}=\pi /2}$ or $0.5{{\bf{e}}}_{Rad}+0.25{{\bf{e}}}_{p=-3,{\varphi }_{0}=\pi /2}-0.25{{\bf{e}}}_{p=5,{\varphi }_{0}=\pi /2}$, respectively. In the latter case, the peripheral part of the generated light pattern is azimuthally polarised and the central part of the pattern has a hybrid polarisation state, partially radial and partially circular. When *φ*_2_ = *π*, the azimuthal polarisation is substantial.

**Figure 5:**
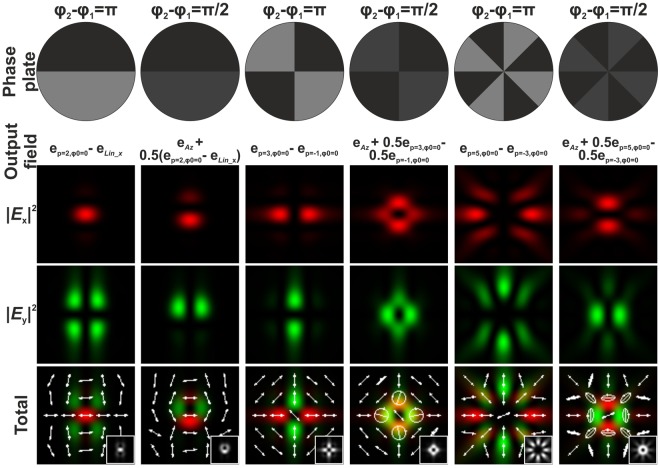
Transformation of the initial azimuthally polarised laser beam passed through different binary multi-sector phase plates. In fact, the generated output fields are superpositions of different CVBs. The distribution of the various components of the electric field in the focal plane of a focusing system (NA = 0.99) are shown. The insets in the total field distribution images show the generated grayscale intensity distributions. The white arrows show the schematic polarisation distributions.

Analogous modelling results obtained when using a light field with azimuthal polarisation as the initial field are shown in Fig. 5. The results are similar to those obtained in the case of the radial polarisation with the exception of a 90 degrees rotation of vectors and the absence of the longitudinal *z*-component. In these cases, it is necessary to use Eq. (8), from which it follows that multi-sector phase plates correspond to the sine functions of multiple angles and lead to a superposition of CVBs in the form of ${c}_{1}{{\bf{e}}}_{Az}+{c}_{2}({{\bf{e}}}_{p=(m+1),{\varphi }_{0}=0}-{{\bf{e}}}_{p=-(m-1),{\varphi }_{0}=0})$, where coefficients *c*_1_ and *c*_2_ depend on the phase difference *φ*_2_ − *φ*_1_ (*c*_1_ = 0 for *φ*_2_ − *φ*_1_ = *π*). For ${{\bf{e}}}_{p=(m+1),{\varphi }_{0}=0}$ and ${{\bf{e}}}_{p=-(m-1),{\varphi }_{0}=0}$, the longitudinal components of the generated light field in the focal plane are equal, therefore this component is absent in the superposition. At small numerical apertures (for example, for NA = 0.3 used for further experimental verification), the contribution of the longitudinal component is also insignificant in other cases, and the difference between the results obtained for an initial radial or azimuthal polarisation is only in the rotation of the light pattern in the focal plane.

### Experiments

**Figure 6:**
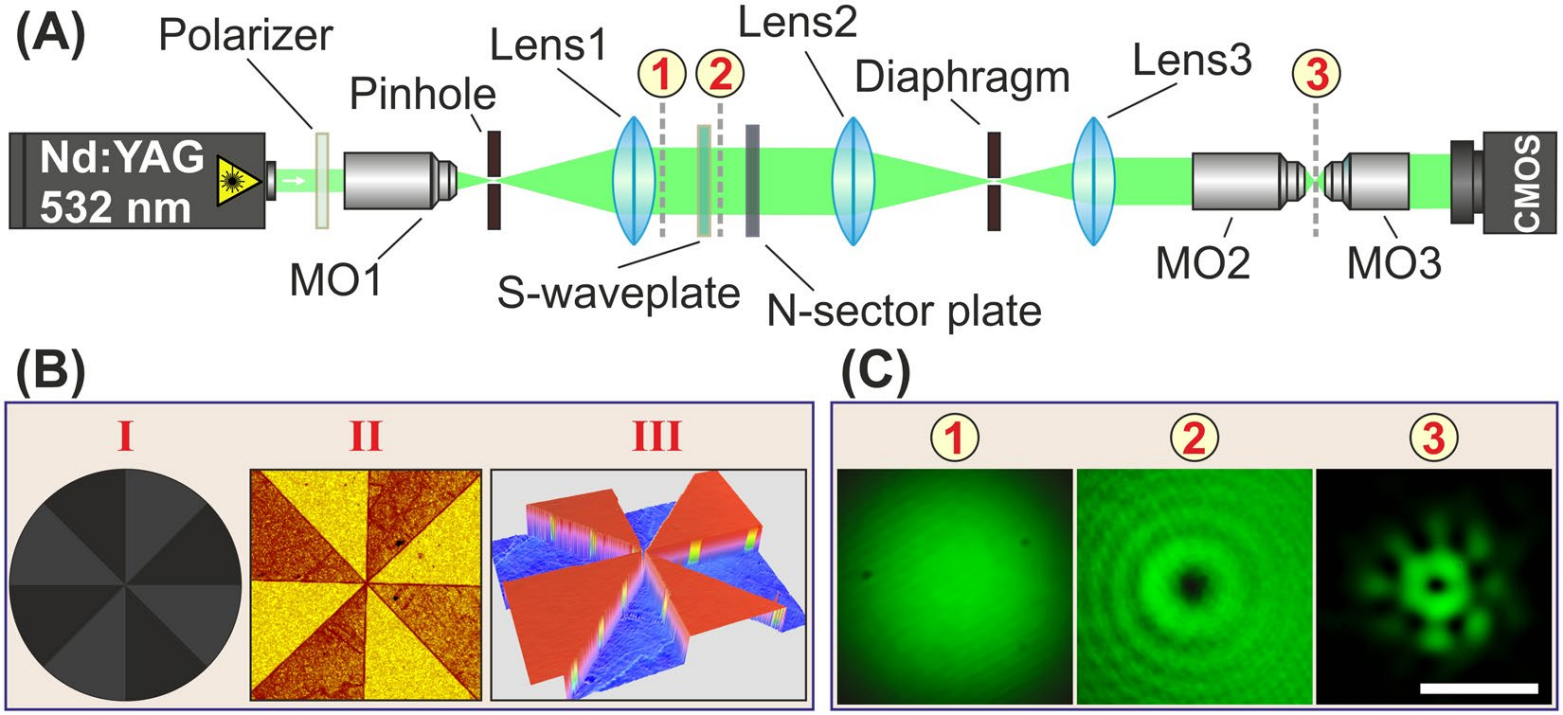
Experimental investigation of polarisation transformation. (**A**) Experimental setup utilised for generation of higher-order CVBs. (**B**) Example of the utilised 8-sector phase plate: (I) designed phase profile with *φ*_2_ − *φ*_1_ = *π*/2, (II) optical microscopy and (III) optical profilometry image of the central part of the manufactured element. (**C**) Beam intensity distributions measured in different planes of the experimental setup. The scale bar is 500 *μ*m.

The optical setup for the experimental investigation of the higher-order hybrid CVBs generation and polarisation transformation is shown in Fig. 6A. The input laser beam was extended and spatially filtered by a system composed of a microobjective MO1 (10×, NA = 0.2), a pinhole PH (aperture size of 40 *μ*m), and a lens L1 (focal length of 150 mm). The collimated linearly polarised laser beam with a Gaussian profile of intensity distribution (waist diameter is approximately 3 mm) was transformed into a “donut”-shaped first-order radially/azimuthally polarised laser beam using a commercially available S-waveplate (Altechna, clear aperture diameter of 4 mm). Then, a wavefront of the formed laser beam was modulated using the fabricated 2, 4, or 8-sector phase plate. The 2, 4, and 8-sector phase plates with a diameter of 4 mm were manufactured on surfaces of 2-mm thick fused silica plates. Two variants of each of the sector plates, with a height difference between neighbouring sectors of approximately 290 ± 20 and 580 ± 20 nm corresponding to *π*/2 and *π*-phase shift at 532 nm, were manufactured (see an example of the manufactured 8-sector phase plate with relief steps with the height *h* = 580 ± 20 nm and side-wall inclination angle of 5 ± 1 degrees in Fig. 6B). A combination of two lenses, L2 (*f*_2_ = 250 mm) and L3 (*f*_3_ = 150 mm), and a diaphragm was used for spatial filtering of the modulated laser beam. Finally, the generated higher-order hybrid CVB was focused by microobjective MO2 (40×, effective numerical aperture NA_eff_ = 0.3) and imaged by microobjective MO3 (100×, NA = 0.8) onto the sensor of the CMOS-video camera (ToupCam, 3328 × 2548 pixel resolution).

**Figure 7:**
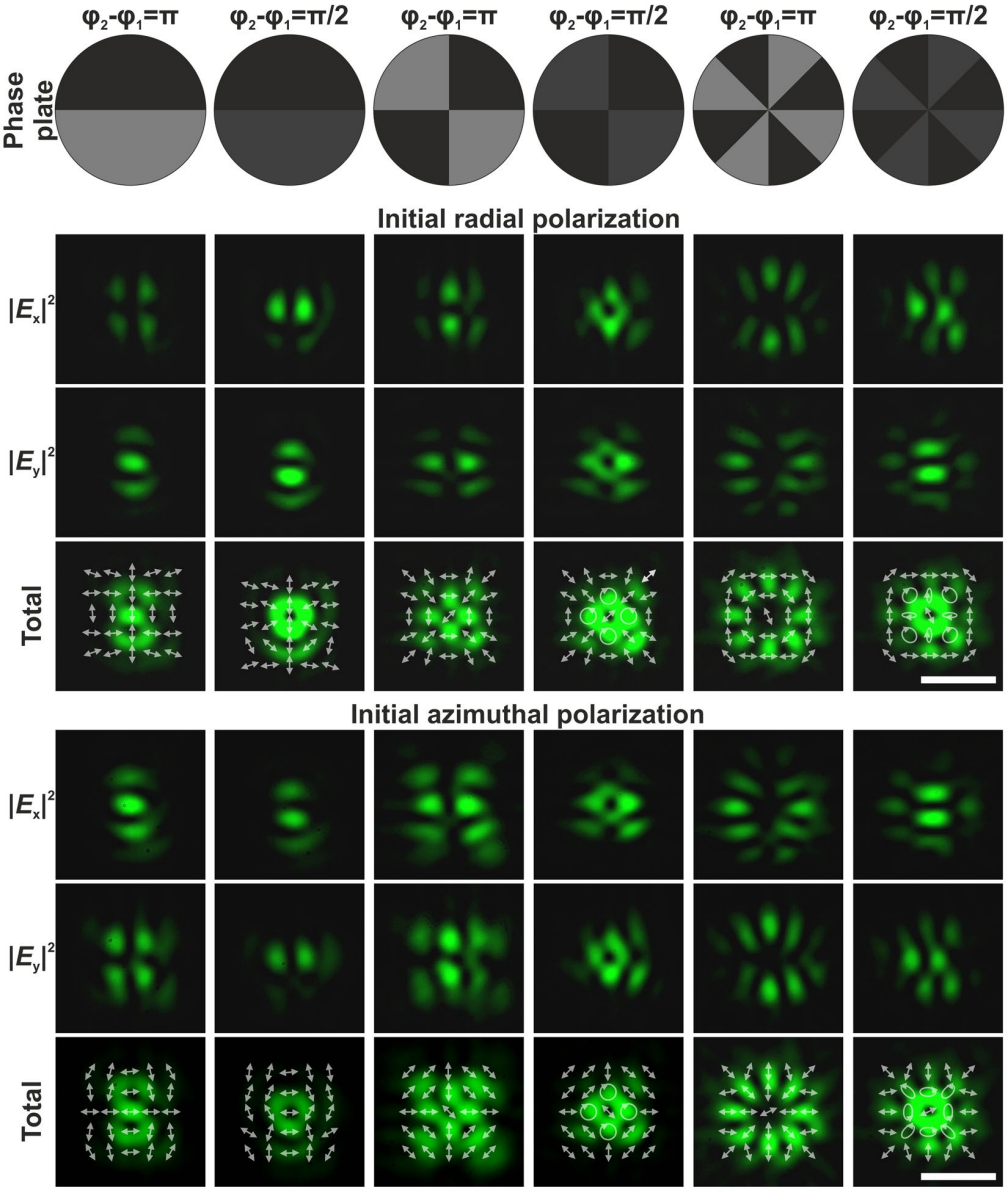
Experimentally obtained transverse component distributions of the focused radially/azimuthally polarised laser beam passed through different binary multi-sector phase plates. The intensity distributions were formed in the focal plane of a microobjective with NA_eff_ = 0.3. The arrows show the schematic polarisation distributions. The scale bar is 500 *μ*m.

Figure 7 shows intensity distributions for different components of the generated hybrid CVBs. A polariser was utilised for analysing the separated transverse electromagnetic field components. Despite the fact the obtained experimental pictures completely coincide with the transverse component distributions obtained in the numerical modelling, the total intensity distributions were different, which may be explained by the difference in the NA values used in the modelling and experiment. It is obvious that the longitudinal component of the electromagnetic field has a noticeable contribution to the formed intensity distribution only under sharp focusing conditions (NA > 0.7). Thus, the obtained experimental results have confirmed the possibility of generating and transforming hybrid CVBs with the help of binary phase elements, even in the case of weak focusing. The generated polarisation states are valid near the optical axis in the focal region. The experimental generation of the patterns in the paraxial case (NA = 0.3) guarantees the preservation of this distribution at the depth of the focus.

## Conclusion and Discussion

We conducted a theoretical analysis of the generation of hybrid cylindrical polarisation states of the light field using optical elements having a transmission function defined by the cosine or sine function of the azimuthal angle in the polar coordinate system. Easy-to-manufacture binary phase elements in the form of the multi-sector phase plate allowed the approximation of these functions and to experimentally realise the polarisation conversion from low-order radially/azimuthally polarised beams into higher-order superpositions. Such transformation does not depend on the numerical aperture of the focusing optical system and can also be performed under conditions of weak focusing (NA < 0.7). In the latter case, the difference between the results obtained for the transformation of low-order radial or azimuthal polarisation is only in the rotation of the light pattern in the focal plane. The experimental results obtained with the help of multi-sector phase plates manufactured on surfaces of fused silica substrates are in good agreement with the numerical modelling results, providing evidence of the proposed technique for the generation of hybrid cylindrical vector beams.

The proposed method evidently does not allow one to generate any polarisation state as it can be done with micro-structured *q*-plates. However, in our opinion, the main advantage of the described polarisation transformation approach in comparison with well known techniques for the generation of higher-order CVBs using structured *q*-plates or single/double spatial light modulators supporting implementation of multi-level phase functions is the simplicity of the transmission function of the binary multi-sector phase elements. Due to this, such elements can be realised not only as ‘static’ phase plates, but also with the help of low cost binary spatial light modulators with low resolution for a higher frame rate for fast switching between different polarisation states. In addition, the utilised multi-sector phase plates have a high-damage threshold which allows the use of these plates with a high power laser.

## Methods

### Binary multi-sector phase plate manufacturing process

A technological process comprising lithography and plasma etching was utilised to manufacture the DOEs. This process consists of the following steps:

1. Hardmask’s direct laser writing in the chromium thin film (45 nm) on the fused silica substrate (UV Fused Silica (JGS3)) produced by circular laser writing system CLWS-200S (Del Mar Photonics, Inc.). The chromium thin film exposed by focused laser radiation oxidises into Cr_2_O_3_. Planar resolution is 1 *μ*m.
2. Unexposed chromium is removed using a hexacyanoferrate (III) potassium (K_3_[Fe(CN)_6_]) solution within 5 minutes.
3. Transfer of the DOE’s profile to the silica substrate achieved using an automated reactive-ion/plasma etching system Caroline PE15 (Ru-Vem Ltd.) through hardmask, with SF_6_ as the working gas and the etching mode parameters, P = 8 · 10^−4^ Pa and gas consumption ${{\rm{Q}}}_{S{F}_{6}}$ = 1.5 l/h.
4. Removal of the Cr_2_O_3_ mask in a solution of K_3_[Fe(CN)_6_] within 1 hour.

### Optical characterization of the manufactured plates

To check the quality, high-precision three-dimensional optical profilometry (NewView 7300, Zygo, vertical resolution <0.1 nm, lateral resolution of 0.36 *μ*m, scan speed of 40 *μ*m/sec) scanning was undertaken, indicating the uniform height distribution for all manufactured multi-sector binary phase plates.
